# The prevalence of functional disability and its impact on older adults in the ASEAN region: a systematic review and meta-analysis

**DOI:** 10.4178/epih.e2022058

**Published:** 2022-07-12

**Authors:** Phei Nie Yau, Clairen Jia Ern Foo, Nicholas Li Jie Cheah, Kar Foong Tang, Shaun Wen Huey Lee

**Affiliations:** 1 School of Pharmacy, Monash University Malaysia, Bandar Sunway, Malaysia; 2 School of Pharmacy, Taylor’s University, Subang Jaya, Malaysia

**Keywords:** Aged, Activities of daily living, Functional status, ASEAN

## Abstract

**OBJECTIVES:**

Functional disability is a common consequence of the ageing process and can lead to poor health outcomes due to the inability to perform activities of daily living (ADL) and instrumental activities of daily living (IADL) independently. However, the prevalence of functional disability among older adults in the Association of Southeast Asian Nations (ASEAN) region is poorly documented. This study aimed to assess the prevalence of functional disability and its impact on older adults in the ASEAN region.

**METHODS:**

A systematic literature search was performed on 4 databases from inception until March 2021 to identify studies examining individuals aged 60 years and above reporting functional disabilities in the ASEAN region. Information on the prevalence and impact of functional disability was extracted, assessed for bias, summarised, and analysed using a random-effects meta-analysis.

**RESULTS:**

Thirty-four studies with 59,944 participants were included. The pooled prevalence of ADL disability was 21.5% (95% confidence interval [CI], 16.2 to 27.3) and that of IADL disability was 46.8% (95% CI, 35.5 to 58.3). Subgroup analyses showed higher prevalence among those of advanced age and women. Adverse impacts included increased years of life with disability and poor health-related quality of life.

**CONCLUSIONS:**

Nearly a quarter of the older adult population in the ASEAN region experience functional disability. These findings highlight the need for further research on the burden and impact of functional disability within this region to allow decision-makers to gauge the severity of the issue, develop policies to reduce the risk of developing functional disabilities, and foster healthy ageing.

## INTRODUCTION

Globally, people are living much longer than they used to due to increasing life expectancy. In tandem with a low birth rate, this is expected to lead to an increasing number of older adults. Population ageing has significant implications for health. While many older adults live healthy lives and contribute to their communities and societies as a whole, functional disability is increasingly reported among older adults. This is most commonly measured based on how well older adults can perform their activities of daily living (ADL), such as getting dressed, walking across a room, bathing, and instrumental activities of daily living (IADL) such as preparing a hot meal, shopping for groceries, or taking medications [1].

The magnitude of functional disability is a key indicator for measuring disease burden and mortality and morbidity rates among older adults [2]. Although functional disability is an important public health issue, there are still insufficient data on its prevalence among older adults, especially in low-income and middle-income countries. The prevalence of at least 1 ADL or IADL disability among older adults aged 60 years and above based on a national survey was reported to be around 36.2% and 37.1% in the United States [3]. In Europe, the prevalence of at least 1 ADL disability ranged between 11% and 44%, and the prevalence of at least 1 IADL disability ranged between 8% and 40% [4]. In 2 examples of rapidly ageing countries, the proportion of older adults aged 65 years and above with disability and requiring long-term care in Japan was reported to be around 20%, while the proportion of older adults with ADL disability in China was 18.3% in 2015 [5,6].

Current evidence on functional disability prevalence is inconclusive as to whether disability rates are rising or declining. Although a decline in prevalence has been reported in some countries [7,8], population ageing can be expected to lead to increasing numbers of older adults with functional disabilities. According to the Association of Southeast Asian Nations (ASEAN) Population Forecast, the percentage of the ageing population in the ASEAN region will double over the next 2 decades, rising from 7.73% in 2015 to 15.49% in 2035 [9]. However, functional disabilities among older adults in the ASEAN region are not well documented despite the rapid demographic transition within ASEAN countries towards becoming aged societies. Given the increased healthcare and long-term care needs among older adults living with functional disabilities, there is a need for a detailed assessment of the prevalence of functional disability and its impact on this population in the ASEAN region.

This systematic review aimed to assess the prevalence of functional disability and its impact on older adults in the ASEAN region. The findings would provide a comprehensive overview of functional disability among older adults, the magnitude and impact of which remain unclear in this region. This evidence can serve as baseline information on the state of functional disability and its severity to inform health and social policy decisions and improve the monitoring of health status of the older adult population. The findings of this study can also assist policymakers in planning policies and allocating resources for care provision and support for older adults to promote healthy ageing and delay the occurrence of functional disabilities.

## MATERIALS AND METHODS

### Search strategy and information sources

Three authors (CJEF, NLJC, and PNY) conducted a literature search of PubMed, Embase, CINAHL, and PsycINFO from database inception until January 31, 2022. Additional relevant studies were identified through hand searches from reference lists. The search terms included “older adults,” “functional disability,” “activities of daily living,” and “ASEAN.” A full list of the keywords and the search strategy are available in Supplementary Material 1. The review was registered on PROSPERO (CRD42021236510).

### Eligibility criteria

Studies were included if they recruited older adults aged 60 years and above who lived in the ASEAN region (Brunei, Cambodia, Indonesia, Lao People’s Democratic Republic, Malaysia, Myanmar, Philippines, Singapore, Thailand, and Vietnam), and reported the prevalence and/or the impact of functional disability measured through self-report or scales/measurement tools. Technical reports, letters, and review articles were excluded.

### Study selection and data extraction

Study titles and abstracts were screened by 3 independent reviewers (CJEF, NLJC, and PNY) based on the inclusion and exclusion criteria. Full texts were retrieved and assessed for eligibility. Any disagreements were discussed and resolved by consensus or adjudication by the senior author (SWHL).

Data were extracted using a piloted, standardised data extraction table. The data extracted included study demographics and design, assessment tools for ADL and/or IADL disability and other functional limitations or impairments, and the impact of functional disability. If a study population was reported multiple times, we extracted information from the most complete study with the largest sample size.

### Quality assessment

The quality of individual studies was assessed independently by 3 reviewers using the Newcastle Ottawa Quality Assessment Scale [10]. Studies were assessed in terms of 3 categories: selection of study participants, comparability of the study, and the assessment of outcome measures (Supplementary Material 2). Any disagreements were resolved by consensus.

### Statistical analysis

The primary outcome of interest was the prevalence of functional disability among older adults. Secondary outcomes of interest were the impact of functional disability and other age-related impairments. The results were summarised narratively. To estimate the prevalence of functional disability, a meta-analysis was performed using the Freeman-Tukey double arcsine method, utilising the metaprop package [11]. A random-effects model was used due to the high heterogeneity between populations. Heterogeneity was assessed using Cochran’s Q and I^2^ statistics, with an I^2^ of more than 75% indicating substantial heterogeneity.

Subgroup analyses were performed for measurement tools and participant type (community-dwelling, older adults recruited from outpatient and primary care settings and hospitalised/institutionalised participants). Studies that reported prevalence by age group (60-74 vs. 75 years and above) and gender were analysed separately. A sensitivity analysis was performed by excluding studies with fewer than 500 participants and studies published in 2009 or earlier. Publication bias was assessed using the Egger test, with p-value < 0.05 indicating significant bias. All analyses were conducted using Stata version 16 (Stata Corp., College Station, TX, USA). Prevalence was computed from eligible studies and illustrated on a map of the ASEAN region.

### Ethics statement

As the current study was a systematic review, ethical approval was not sough since no human or animal intervention was performed.

## RESULTS

**Figure 1.**
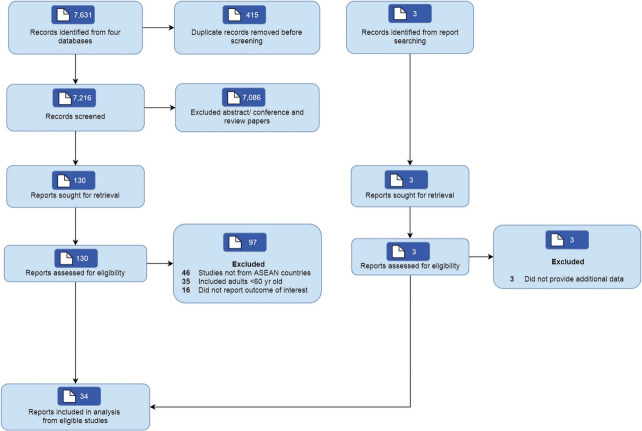
PRISMA (Preferred Reporting Items for Systematic Reviews and Meta-Analyses) flow diagram of study selection. ASEAN, Association of Southeast Asian Nations.

The search identified 7,631 articles, and 7,501 were excluded after title and abstract screening. A total of 39 articles describing 34 studies with 59,944 participants from 6 ASEAN countries were included in the review (Figure 1), as data from 3 cohorts were reported in several publications [12-19]. Twenty-five studies were included in the meta-analysis [14,17,18,20-41].

### Study characteristics

There were 28 cross-sectional [14,18,20-28,30,31,33-35,38-49] and 5 cohort studies [17,29,32,37,50] (Supplementary Material 3). One study reported findings based on a cross-sectional and a cohort study [36]. These studies originated mostly from Singapore (n = 10), Malaysia (n = 9), and Thailand (n = 7). These studies evaluated functional disability using validated ADL and IADL assessment tools, with language modifications and adaptations. Functional disability was defined as the need for assistance or inability to perform ADL or IADL. Of the 27 studies reporting ADL, 15 studies used the Barthel Index [14,17,20-22,24,28,29,32,34-36,43,48,50], while several studies reported using the Katz scale [22,28,29,33,39,42,44] and other ADL assessments. The Lawton and Brody scale was used in 14 studies to assess IADL [14,17,26,27,30-32,34,39,41-44,49]. Of the 33 studies with data on ADL and/or IADL disability, 23 studies also reported vision, hearing, and physical function/mobility impairments. Eleven studies reported data on cognitive impairment, using tools such as the Mini-Mental State Examination (MMSE), Chula Mental Test, and Hodkinson Abbreviated Mental Test.

### Quality assessment

Eleven studies were rated as very good quality, 11 were rated as good, and 12 were rated as satisfactory. Some concerns were noted in the study representativeness domain, as most studies did not describe and/or achieve a satisfactory response. Of the 34 studies, 3 did not fully describe the statistical analysis methods (Supplementary Material 4).

### Overall prevalence of functional disability

#### Activities of daily living disability

**Figure 2.**
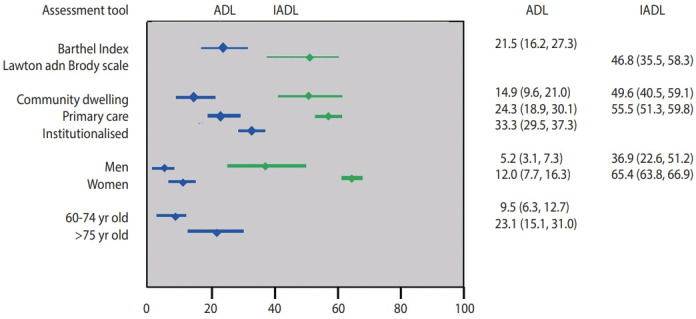
Prevalence of activities of daily living (ADL) and instrumental activities of daily living (IADL) disabilities and subgroup analyses by an assessment tool, participant type, gender, and age group. Valeus are presented as % (95% confidence interval).

**Figure 3.**
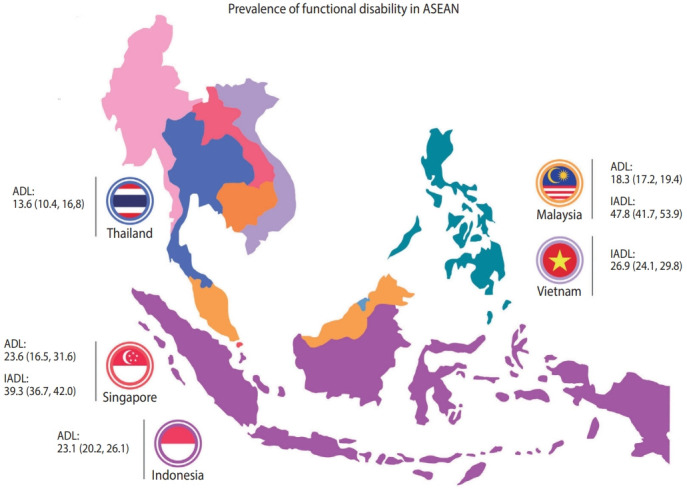
Prevalence of activities of daily living (ADL) and instrumental activities of daily living (IADL) disabilities in the Association of Southeast Asian Nations (ASEN) region. Values are presented as % (95% confidence interval).

Twenty-three studies reported the prevalence of ADL disability. Bathing and incontinence/bladder control were the most impaired ADL component reported in all studies. In general, studies showed that the prevalence of ADL disability increased with age and was higher in women and, to a lesser extent, in those with multimorbidity. The overall prevalence of ADL disability in 13 studies using the Barthel Index (representing 13,127 older adults in the ASEAN region) was 21.5% (95% confidence interval [CI], 16.2 to 27.3; Figures 2 and 3). The highest prevalence of ADL disability was noted in Singapore, with a prevalence of 55.1%, while the lowest was in Thailand (7.2%). Subgroup analyses showed that participants recruited from the community had the lowest prevalence of ADL disability (14.9%), followed by older adults recruited from outpatient/primary care settings (24.3%), with the highest prevalence reported among hospitalised and institutionalised participants (33.3%) [36,37]. In studies that reported prevalence by age, the prevalence of ADL disability was also higher in those aged 75 years and above (23.1%) than in their younger counterparts aged 60-74 years (9.5%). Five studies also reported prevalence by gender and showed that the prevalence of ADL disability was 2.3 times higher in women than in men (12.0 vs. 5.2%; p< 0.01) [14,18,23,27,33].

#### Instrumental activities of daily living disability

Among studies that reported the prevalence of IADL components, food preparation, grocery shopping, and ability to use the telephone were the most commonly impaired activities. IADL limitations were reported to be associated with increasing age, women gender, comorbidities, illiteracy, education and social support [14,26,27,32,39]. The overall prevalence of IADL disability among 7,311 older adult participants in the ASEAN region using the Lawton and Brody scale was 46.8% (95% CI, 35.5 to 58.3; Figures 2 and 3). Similar to ADL disability, the highest prevalence of IADL disability was noted in Singapore, with a prevalence of 76.0%, while the lowest was in Vietnam (23.9%). Subgroup analyses by assessment tool, gender, and participant type were also conducted. Three studies reported the prevalence of IADL disability by gender, with a higher prevalence reported among women (65.4%) than among men (36.9%, p< 0.01) [14,27,33]. The prevalence of IADL disability was relatively similar among communitydwelling participants (49.6%) and older adults recruited from outpatient/primary care settings (55.5%). As only 1 study recruited institutionalised older adults and reported data on IADL, this information was not pooled in our analysis [39].

### Sensitivity analysis

A sensitivity analysis of the effect of sample size on the prevalence of ADL and IADL disabilities showed a lower pooled prevalence for ADL and IADL disabilities with studies of larger sample size (n> 500) (Table 1). A sensitivity analysis for studies conducted from 2010 onwards showed similar pooled prevalence rates for both ADL and IADL disabilities.

### Secondary outcomes

#### Physical limitations

Three studies reported mobility limitations due to functional disability, the prevalence of which ranged from 14.8% to 50.9% [37,46,48]. Women participants [22,41], those with vision or hearing impairment [38], and older participants (70-79 years) [33] more frequently experienced higher physical limitations.

#### Vision and hearing impairment

The prevalence of visual impairment among older adults ranged from 11.2% to 85.1%. There was a trend for poorer visual acuity with advancing age (70 years and above). Seven studies also reported the prevalence of hearing impairment among older adults, ranging from 5.5% to 26.9% [25,32,38-40,45,46].

#### Cognitive impairment

Several studies also reported the presence of cognitive impairment, with a prevalence ranging from 3.5% to 64.0%. Similar to ADL and IADL disabilities, age was found to be a predictor of cognitive impairment. The prevalence of cognitive impairment was reported to be higher for women than for men regardless of age group (60-69 or 70-79 years) [33] and in frail individuals than in robust individuals [30].

#### Impact of functional disability

Due to ADL or IADL disability, participants were reported to have an increased risk of rehospitalisation [29], more years lived with disability [23,40], frailty [36], and poor health-related quality of life [34]. Older adults with balance (mobility) impairment were more likely to have an increased fear of falling, leading to activity restriction [48]. Age-related vision impairment was reported to increase the risk of developing ADL, IADL, and mobility disability by 3-fold to 5-fold [44]. In a study conducted in Malaysia [45], vision and hearing loss were associated with lower cognitive function, where older adults with these impairments had significantly lower MMSE scores than those without impairments. Older adults with vision and/or hearing impairment were also more likely to live more years with ADL or physical limitations [38].

## DISCUSSION

In this review, we found that functional disability affects a large number of older adults resident in the ASEAN region. In particular, approximately 16.0 million older adults lived with ADL disability and 34.8 million older adults lived with IADL disability in 2020 in the ASEAN region [51,52]. This figure is expected to increase to 37.8 million older adults living with ADL disability and 82.4 million older adults living with IADL disability in 2050, in line with the rapidly ageing population [52]. The results from our analysis showed that the prevalence of ADL or IADL disability was consistently higher in adults of advanced age and among older women. This is similar to findings reported in the literature, where disability trends were observed to be dependent on age and gender [53-55]. These parameters could be used as predictors for functional decline in the ageing population, as well as a means of measuring and targeting at-risk groups for intervention, especially those who are institutionalised.

Examining prevalence by country, we noted that the prevalence of ADL and IADL disabilities will likely impact Singapore and Thailand the most, given the large ageing population in both countries [51,52]. However, there are currently insufficient data for the identification of ADL and IADL disabilities by more specific geographical patterns—that is, to determine whether these disabilities are more likely to impact those living in rural compared to urban areas or in high-income compared to middle-income or low-income countries. Indeed, our review only identified that there are limited data from other ASEAN countries such as Brunei, Cambodia, Myanmar, and the Philippines. This information, if it becomes available, could provide important insights for policymakers, as it would help them plan for healthcare services in these areas.

The prevalence of functional disability among older adults differs among countries but is expected to increase in parallel with global population ageing. Given the unfavourable impact of agerelated functional impairments, an early screening or assessment of functional impairment/disability is vital, especially at the primary care level [16], to ensure that timely interventions can be started to delay the occurrence or progression of functional disability or age-related impairments. There is a need for comprehensive geriatric assessments in primary care and hospital settings to identify patients at risk of functional disabilities, such as older adults with frequent falls or hospitalisation episodes, multimorbidity, cognitive decline, and polypharmacy.

To our best knowledge, this is the first review investigating the prevalence of functional disability and its impact among older adults in the ASEAN region. This review also gathered data on ageing-related disabilities, namely visual, hearing, mobility, and cognitive impairment. Further research is needed to better understand the severity and impact of functional disability among older adults in ASEAN countries. Older adults who develop ADL/IADL disabilities might eventually live with a high disability burden and have an increased reliance on informal care [56] or a need for healthcare, acute care, rehabilitation, or long-term care services. This highlights the need for better investment and policymaking for healthcare and long-term care systems to address the growing disability burden and meet the needs of the ageing population. In line with the United Nations Decade of Healthy Ageing initiative, evidence on the prevalence of functional disability would be helpful for decision-makers in determining indicators and regional estimates to support integrated care and long-term care for older adults [57,58].

There was a vast discrepancy in the prevalence reported due to the different scales/measurement tools used, cut-off points for disability, and the inclusion and exclusion criteria set in each study. As such, we could not ascertain causal relationships. High heterogeneity was observed, as in most meta-analyses of prevalence. Some studies classified their study subjects into different subgroups, such as those with concurrent sensory and cognitive impairments, which had higher prevalence rates due to the presence of more functional decline. There was insufficient information to identify geographical patterns, and the lack of longitudinal cohort studies and repeated national surveys also prevented the assessment of trends in functional disability. We only included articles in English or those translated into English and thus might have missed some relevant studies.

## CONCLUSION

In summary, functional disability was found to be prevalent among older adults in the ASEAN region, with a pooled prevalence of 22% and 47% for ADL and IADL disabilities, respectively. These findings also highlight that functional disability was more prevalent among older adults of advanced age and women. Challenges remain in interpreting prevalence across the ASEAN region due to limited data and considerable evidence of heterogeneity. More research is needed to estimate the burden of disability and understand its impact on older adults within the ASEAN region. Evidence on the prevalence of functional disability would be useful to inform health and social care decision-making, improve health promotion and care to reduce the risk of functional disability, and drive efforts towards fostering healthy ageing as part of the United Nations Decade of Healthy Ageing initiative.

## Figures and Tables

**Table 1.** Sensitivity analysis of the effect of sample size and study year

Variables	No. of studies (sample size)	Prevalence, % (95% CI)
ADL disability		
Sample size: n>500	4 (10,155)	13.0 (7.3, 19.9)
Year 2010 onwards	8 (7,521)	22.8 (18.9, 26.9)
IADL disability		
Sample size: n>500	3 (5,705)	30.6 (35.5, 58.3)
Year 2010 onwards	8 (7,051)	48.5 (36.1, 61.0)

CI, confidence interval; ADL, activities of daily living; IADL, instrumental activities of daily living.

## SUPPLEMENTARY MATERIALS

Supplementary Material 1. Search strategy

Supplementary Material 2. Newcastle-Ottawa Quality Assessment Scale (adapted for cross-sectional studies)

Supplementary Material 3. Characteristics of included studies

Supplementary Material 4. Quality assessment for included studies using the adapted Newcastle Ottawa Scale.
