# Supplementary material for: The prevalence of functional disability and its impact on older adults in the ASEAN region: a systematic review and meta-analysis
Source: Epidemiol Health. 2022 Jul 12;44:e2022058. doi: 10.4178/epih.e2022058 (PMC9754909; doi:10.4178/epih.e2022058)
Supplement: Supplementary Material 1. — Search strategy [file epih-44-e2022058-suppl1.docx]

**The Prevalence of Functional Disability and Its Impact on Older Adults in ASEAN Region: A Systematic Review and Meta-analysis**

Supplementary Material 1. Search strategy

| # 1 | (aged[tiab]) OR (“aged, 60 and over”[tiab]) OR (aging[tiab]) OR (older[tiab]) OR (elder[tiab]) OR (“older adults”[tiab]) OR (“oldest old”[tiab]) OR (“very old”[tiab]) OR (“very elderly”[tiab]) |
| --- | --- |
| # 2 | functional disability OR functional impairment OR functional deficits OR everyday functioning OR everyday function OR functional performance OR everyday cognition OR functional ability OR functional abilities OR functional independence OR activities of daily living OR adl OR daily functioning OR everyday skills OR daily living skills OR instrumental activities of daily living OR iadl OR independent living OR complex activities of daily living OR complex adl OR advanced activities of daily living OR advanced adl |
| #3 | ASEAN OR Brunei OR Thailand OR Laos OR Lao PDR OR Singapore OR Malaysia OR Philippines OR Vietnam OR Viet Nam OR Myanmar OR Cambodia OR Indonesia |
| #4 | #1 AND #2 AND #3 |
