# Supplementary material for: The prevalence of functional disability and its impact on older adults in the ASEAN region: a systematic review and meta-analysis
Source: Epidemiol Health. 2022 Jul 12;44:e2022058. doi: 10.4178/epih.e2022058 (PMC9754909; doi:10.4178/epih.e2022058)
Supplement: Supplementary Material 2. — Newcastle-Ottawa Quality Assessment Scale (adapted for cross-sectional studies) [file epih-44-e2022058-suppl2.docx]

Supplementary Material 2. Newcastle-Ottawa Quality Assessment Scale (adapted for cross-sectional studies)

*Selection: (Maximum 5 stars)*

1. **Representativeness of the sample:**
   1. Truly representative of the average in the target population. * (all subjects or random sampling)
   2. Somewhat representative of the average in the target group. * (non-random sampling)
   3. Selected group of users.
   4. No description of the sampling strategy.
2. **Sample size:**
   1. Justified and satisfactory (including sample size calculation). *
   2. Not justified.
3. **Non-respondents:**
   1. Comparability between respondents and non-respondents’ characteristics is established, and the response rate is satisfactory. *
   2. The response rate is unsatisfactory, or the comparability between respondents and non-respondents is unsatisfactory.
   3. No description of the response rate or the characteristics of the responders and the non-responders.
4. **Ascertainment of the exposure (risk factor):**
   1. Validated measurement tool. **
   2. Non-validated measurement tool, but the tool is available or described. *
   3. No description of the measurement tool.

*Comparability:* *(Maximum 2 stars)*

1. **The subjects in different outcome groups are comparable, based on study design or analysis. Confounding factors are controlled.**
   1. The study controls for the most important factor (select one). *
   2. The study control for any additional factor. *

*Outcome: (Maximum 3 stars)*

1. **Assessment of outcome:**
   1. Independent blind assessment. **
   2. Record linkage. **
   3. Self-report. *
   4. No description.
2. **Statistical test:**
   1. Statistical test used to analyse the data clearly described, appropriate and measures of association presented including confidence intervals and probability level (p value). *
   2. Statistical test not appropriate, not described or incomplete.
