# Supplementary material for: The prevalence of functional disability and its impact on older adults in the ASEAN region: a systematic review and meta-analysis
Source: Epidemiol Health. 2022 Jul 12;44:e2022058. doi: 10.4178/epih.e2022058 (PMC9754909; doi:10.4178/epih.e2022058)
Supplement: Supplementary Material 3. — Characteristics of included studies [file epih-44-e2022058-suppl3.docx]

Supplementary Material 3. Characteristics of included studies

| **Main author (year of publication)** | **Country** | **Study design** | **Year of data collection** | **Number of participants** | **Mean age (SD)** | **Percentage of female (%)** | **Participant characteristics** | **Functional ability assessment** | **Prevalence of functional disability** | **Summary of main results** | **Assessment for other age-related conditions** | **Prevalence of other age-related conditions** | **Summary of results for other age-related conditions** | **Impact of functional disability** |
| --- | --- | --- | --- | --- | --- | --- | --- | --- | --- | --- | --- | --- | --- | --- |
| Pengpid S *et al* (2019) | Indonesia | Cross-sectional | 2014-2015 | 2630 | n/a | 49.7 | Community-dwelling participants aged 60 years and above from the Indonesian Family Life Survey in 2014-2015. | Katz scale | ADL/IADL disability: | 22.5% had one ADL/IADL disability, 6.7% had two or more ADL/IADL disabilities. Out of 214 participants with frailty, 10.9% had one ADL/IADL disability, and 22% had two or more. | Fried’s Frailty Phenotype | Prefrailty: 61.6% | The overall prevalence of frailty and prefrailty was 8.1%, and 61.6%. 7.7% of men and 8.5% of women were frail, and 59.5% of men and 63.7% of women were prefrail. | n/a |
|  |  |  |  |  |  |  |  | Lawton and Brody Scale | None: n=1827 (70.5%) |  |  | Frailty: 8.1% |  |  |
|  |  |  |  |  |  |  |  |  | One: n=605 (22.9%) |  |  |  |  |  |
|  |  |  |  |  |  |  |  |  | Two or more: n=198 (6.7%) |  |  |  |  |  |
| Rensa R *et al* (2019) | Indonesia | Cross-sectional | 2017 | 325 | n/a | 100 | Urban community-dwelling older women aged 60 years and above in Rukun Warga 01–09, Kelurahan Kalianyar (Kecamatan Tambora, West Jakarta, Indonesia) and community-based elderly day care facilities. | Barthel Index | BADL independent: n=280 (86.2%) | The proportion of independent subjects was 86.2%. There were 44 subjects (13.5%) who had mild dependency and one with moderate dependency. 28.2% of frail subjects had a score of <20. Decreased functional status (ADL score<20) was significantly associated with frailty syndrome. | Abbreviated Mental Test (AMT) to assess cognitive status | Cognitive status: | The proportion of older women in the robust, pre-frail, and frail categories was 12.6%, 63.4%, and 24%, respectively. 22.5% and 4.3% had mild and severe memory disorders, respectively. | n/a |
|  |  |  |  |  |  |  |  |  | Mild dependency: n=44 (13.5%) |  |  | Normal (8-10): n=238 (73.2%) |  |  |
|  |  |  |  |  |  |  |  |  | Moderate dependency: n=1 (0.3%) |  |  | Mild memory disorder (4-7): n= 73 (22.5%) |  |  |
|  |  |  |  |  |  |  |  |  |  |  | Cardiovascular Health Study (CHS) scoring system to assess frailty | Severe memory disorder (0-3): n=14 (4.3%) |  |  |
|  |  |  |  |  |  |  |  |  |  |  |  | Frailty: |  |  |
|  |  |  |  |  |  |  |  |  |  |  |  | Frail: 24% |  |  |
|  |  |  |  |  |  |  |  |  |  |  |  | Pre-frail: 63.4% |  |  |
|  |  |  |  |  |  |  |  |  |  |  | Physical examination to assess handgrip strength. | Weakness (poor handgrip strength and low physical activity): |  |  |
|  |  |  |  |  |  |  |  |  |  |  |  | Frail (89.7%) |  |  |
|  |  |  |  |  |  |  |  |  |  |  |  | Pre-frail (74.3%) |  |  |
| Setiati S *et al* (2019) | Indonesia | Cross-sectional | n/a | 488 | 72.4 | 59.8 | Patients aged 60 years or above who visited the geriatric outpatient clinic at five hospitals. | Barthel ADL scale | ADL dependent: 147 (32.8%) | 147 (32.8%) participants were ADL dependent (totally dependent, heavily dependent, moderately dependent, and mildly dependent). | Frailty Index-40 item questionnaire  Handgrip and gait speed measurement | Frailty: | 13.2% were robust, 61.6% were prefrail, and 25.2% were frail. Bivariate analysis showed that age (OR 2.83; 95% CI 1.69–4.73), functional status (OR 3.97; 95% CI 2.54–6.20), and polypharmacy (OR 1.82; 95% CI 1.05–3.15) were found to be significantly associated with frailty. | The risk of frailty was 2.9 times greater among older adults with dependent functional status. |
|  |  |  |  |  |  |  |  |  |  |  |  | Robust: n=59 (13.2%) |  |  |
|  |  |  |  |  |  |  |  |  |  |  |  | Pre-frail: n=276 (67.6%) |  |  |
|  |  |  |  |  |  |  |  |  |  |  |  | Frail: n=113 (n=25.2%) |  |  |
|  |  | Prospective cohort study | At 12-month follow-up | 162 | 72.9 | 57.4 |  |  | ADL functional status (ADL-dependent, score<20) | 25.9% of participants were dependent (ADL score<20) at baseline, which increased to 38.3% at 12 months follow up. |  | Frailty state (baseline; at 12 months) | The proportion of frail subjects increased after 12 months; 44 (27.2%) of the subjects had frailty state worsening. Slow gait speed (RR 2.68; 95% CI 1.57–4.57, p < 0.05) is one of the factors associated with frailty state worsening. |  |
|  |  |  |  |  |  |  |  |  |  |  |  | Robust: n=9 (5.6%); n=2 (1.2%) |  |  |
|  |  |  |  |  |  |  |  |  | At baseline: 42 (25.9%) |  |  | Pre-frail: n=114 (70.4%); n=104 (64.2%) |  |  |
|  |  |  |  |  |  |  |  |  | At 12-month follow-up: 62 (38.3%) |  |  | Frail: n=39 (24.1%); n=56 (34.6%) |  |  |
| Soejono CH *et al* (2019) | Indonesia | Retrospective cohort study | 2013-2014 | 158 | n/a | 51.4 (pre-UHCP), 60.5 (UHCP) | Patients with geriatric syndromes hospitalised at Cipto Mangunkusumo Hospital, Jakarta, Indonesia. | Barthel Index | **Only 134 subjects have a complete functional status Pre-UHCP (n=72) | The proportion of participants with mild, moderate, severe and total dependency pre-UHCP (n=72) were 25%, 19.4%, 22.2%, and 25%, while for UHCP (n=86), these were 19.8%, 16.3%, 15.1% and 20.9% respectively. The proportion of those discharged with independent functional status was higher in the UHCP group (from 6.1% to 19.7%) than in the pre-UHCP group (from 2.9% to 7.4%). | n/a | n/a | n/a | n/a |
|  |  |  |  | Pre-universal health coverage program (UHCP) group (hospitalised from July to December 2013): 72 subjects |  |  |  | *independent (score 20) | Independent: 2 (2.8%) |  |  |  |  |  |
|  |  |  |  |  |  |  |  | *mildly dependent (score 12-19) | Mildly dependent: 18 (25%) |  |  |  |  |  |
|  |  |  |  |  |  |  |  | *moderately dependent (score 9-11) | Moderately dependent: 14 (19.4%) |  |  |  |  |  |
|  |  |  |  |  |  |  |  | *severely dependent (score 5-8) | Severely dependent: 16 (22.2%) |  |  |  |  |  |
|  |  |  |  | UHCP group (hospitalised from January to June 2014): 86 |  |  |  | *totally dependent (score 0-4) | Totally dependent: 18 (25%) |  |  |  |  |  |
|  |  |  |  |  |  |  |  |  | no data: 4 (5.6%) |  |  |  |  |  |
|  |  |  |  |  |  |  |  |  | UHCP (n=86) |  |  |  |  |  |
|  |  |  |  |  |  |  |  |  | Independent: 4 (4.7%) |  |  |  |  |  |
|  |  |  |  |  |  |  |  |  | Mildly dependent: 17 (19.8%) |  |  |  |  |  |
|  |  |  |  |  |  |  |  |  | Moderately dependent: 14 (16.3%) |  |  |  |  |  |
|  |  |  |  |  |  |  |  |  | Severely dependent: 13 (15.1%) |  |  |  |  |  |
|  |  |  |  |  |  |  |  |  | Totally dependent: 18 (20.9%) |  |  |  |  |  |
|  |  |  |  |  |  |  |  |  | no data: 20 (23.2%) |  |  |  |  |  |
| Nambooze J *et al* (2014) | Lao PDR | Cross-sectional | 2012 | 144 | n/a | 61.8 | Community-dwelling older adults aged 65 years and above in three rural ethnic groups, the Oy, the Brau and the Lao, of southern Lao PDR. | Barthel ADL scale | Limitations in ADL: | 47.2% of the respondents from the Oy, 43.9% from the Brau and 20% from the Lao had limitations in ADL, whereas 98.1% of Oy respondents, 97.6% of Brau respondents and 86% of Lao respondents had limitations in IADL. The mean ADL scores were 16.9 ± 2.1, 18.6 ± 0.5 and 17.8 ± 1.6 for the Oy, Brau and Lao, respectively, while the mean IADL scores were 7.0 ± 1.8, 7.2 ± 2.1 and 6.9 ± 2.2, respectively. Most respondents had limitations in IADL, especially shopping, phone usage and handling finances. | n/a | n/a | n/a | n/a |
|  |  |  |  |  |  |  |  | Lawton and Brody IADL questionnaire. IADL questionnaire consisted of 6 items as housekeeping & laundry were combined into 1 question, and responsibility for own medication was omitted | Oy respondents: 47.2% |  |  |  |  |  |
|  |  |  |  |  |  |  |  |  | Brau respondents: 43.9% |  |  |  |  |  |
|  |  |  |  |  |  |  |  |  | Lao respondents: 20% |  |  |  |  |  |
|  |  |  |  |  |  |  |  |  | Limitations in IADL: |  |  |  |  |  |
|  |  |  |  |  |  |  |  |  | Oy respondents: 98.1% |  |  |  |  |  |
|  |  |  |  |  |  |  |  |  | Brau respondents: 97.6% |  |  |  |  |  |
|  |  |  |  |  |  |  |  |  | Lao respondents: 86% |  |  |  |  |  |
| Falahaty K *et al* (2015) | Malaysia | Cross-sectional | 2012 | 150 | 69.39 ±7.31 | 54 | Older persons aged 60 years and above in two welfare homes in Malaysia. | Katz ADL Index | Difficulties in performing ADL: | Most reported ADL difficulty for bathing (59.3%) For IADL, most reported difficulties for food preparation (88.0%). | Snellen E Chart | Visual acuity impairment: 46% (n=69) | Blindness increased significantly with age from 5.8% in subjects 60-69 years of age and 25.8% in subjects 70-79 years of age to 67.7% in subjects 80 years old or older. Visual impairment also increased with age ranging from 34.6% in subjects aged 60-69 to more than 71% in subjects aged 70-79 | Visual impairment increases the risk for ADL, IADL and mobility limitation three- to five-fold. Visually impaired persons experience many restrictions in daily life due to vision loss. |
|  |  |  |  |  |  |  |  | Lawton-Brody IADL scale | Bathing 59.3% (n=89) |  |  |  |  |  |
|  |  |  |  |  |  |  |  |  | Feeding 55.3% (n=83) |  |  | Blindness: 28% (n=42) |  |  |
|  |  |  |  |  |  |  |  |  | Continence 54.7% (n=82) |  |  |  |  |  |
|  |  |  |  |  |  |  |  |  | Transferring 47.3% (n=71) |  |  |  |  |  |
|  |  |  |  |  |  |  |  |  | Dressing 41.3% (n=62) |  |  |  |  |  |
|  |  |  |  |  |  |  |  |  | Toileting 31.3% (n=56) |  |  |  |  |  |
|  |  |  |  |  |  |  |  |  | Difficulties in performing IADL: |  |  |  |  |  |
|  |  |  |  |  |  |  |  |  | Food preparation 88.0% (n=102) |  |  |  |  |  |
|  |  |  |  |  |  |  |  |  | Housekeeping 66.0% (n=99) |  |  |  |  |  |
|  |  |  |  |  |  |  |  |  | Use transportation 60.0% (n=90) |  |  |  |  |  |
|  |  |  |  |  |  |  |  |  | Shopping 58.7% (n=88) |  |  |  |  |  |
|  |  |  |  |  |  |  |  |  | Laundry 58.7% (n=88) |  |  |  |  |  |
|  |  |  |  |  |  |  |  |  | Responsibility for own medications 54.0% (n=81) |  |  |  |  |  |
|  |  |  |  |  |  |  |  |  | Ability to handle finances 53.3% (n=80) |  |  |  |  |  |
|  |  |  |  |  |  |  |  |  | Ability to use telephone 33.3% (n=50) |  |  |  |  |  |
| Hairi NN *et al* (2010) | Malaysia | Cross-sectional | 2007-2008 | 765 | 69 ± 7 | 62.6 | Rural community-dwelling older people aged 60 years and above from the Alor Gajah Older People Health Survey (AGOPHS). | 10-item Barthel Index | Prevalence of physical disability and functional limitation (n=738) | 24.7% reported needing help in at least one of the 10 ADLs in the Barthel Index. The prevalence of disability based on at least one item of the six-item ADL scale was 14.4%, and the prevalence of disability based on at least one item in the five ADL scale was 10.6%. Overall prevalence of ADL disability increased with advancing age. The prevalence of needing help in at least one of the ten ADLs of the Barthel Index increased from 6% in those aged 60-64 years to 50% of those aged 75 years and older. | Elderly Cognitive Assessment Questionnaire (ECAQ) | Cognitive impairment:  Normal cognition: n=659 (86.1%); borderline impaired: n=79 (10.4%); probably cognitively impaired: n=27 (3.5%) | The prevalence of functional limitation was 19.5%. The prevalence of functional limitation rose from 6% in those aged 60 to 64 years to 48% in the 75 and above. Prevalence of self-reported physical disability and objective measurement of functional limitation was higher in women than in men. | n/a |
|  |  |  |  |  |  |  |  | 6-item Katz ADL scale (feeding, dressing, bathing, toileting, transferring and walking) | 24.7% reported needing help in at least one of the 10 ADLs in the Barthel Index |  | Snellen E Chart |  |  |  |
|  |  |  |  |  |  |  |  | 5-item ADL scale (feeding, dressing, bathing, toileting and transferring) | 14.3% disability based on at least one item of the 6 -item Katz ADL scale |  | Tinetti Performance Oriented Mobility Assessment Tool to measure functional limitation (gait and balance) | Visual impairment: |  |  |
|  |  |  |  |  |  |  |  |  |  |  |  | Normal visual acuity: n=579; mild to moderate visual acuity: n=126; blind: n=33 |  |  |
|  |  |  |  |  |  |  |  |  | 10.6% disability based on at least one item in the 5-item ADL scale |  |  | Prevalence of functional limitation was 19.5% |  |  |
| Harithasan D *et al* (2020) | Malaysia | Cross-sectional | 2013 | 229 | n/a | 53.3 | Community-dwelling older adults aged 60 years and older in Selangor state. | n/a | n/a | n/a | Cognitive function: Malay version of MMSE | Dual sensory impairment (DSI): 19 (8.3%) | The no impairment group had the highest mean score for cognitive score, followed by vision loss only, hearing loss only, and DSI groups. Bonferroni post-hoc analysis revealed that DSI and hearing loss only groups had significantly lower scores than the no impairment group. | Hearing loss alone and DSI were independently associated with lower cognitive function (reduced MMSE score). DSI was associated with lower quality of life. |
|  |  |  |  |  |  |  |  |  |  |  | Vision: Lighthouse International Chart for literate participants; tumbling E for those unable to read or write | Vision loss only: 27 (11.8%) |  |  |
|  |  |  |  |  |  |  |  |  |  |  |  |  |  |  |
|  |  |  |  |  |  |  |  |  |  |  |  | Hearing loss only: 46 (20.1%) |  |  |
|  |  |  |  |  |  |  |  |  |  |  | Audiometric: otoscopic examination, screening tympanometry, and measurement of air and bone conduction hearing thresholds. |  |  |  |
| Loh KY *et al* (2005) | Malaysia | Cross-sectional | 2001 | 260 | 67.5 (male), 65.5 (female) | 54.6 | Patients aged above 60 from a semi-rural government health clinic | Lawton and Brody IADL scale | 87 individuals had at least one functional disability, prevalence = 33.5%. | Common disabilities were the inability to do shopping without help (40%), followed by difficulty in climbing stairs without assistance (36.6%) and impairment in taking medication (35%). | n/a | n/a | n/a | n/a |
|  |  |  |  |  |  |  |  |  | Two respondents were totally dependent in which they were unable to perform the nine IADL without getting help, the prevalence of total disability = 0.8%. |  |  |  |  |  |
|  |  |  |  |  |  |  |  |  | Make phone calls: 20%  Go out: 25.9%  Grocery shopping: 40%  Prepare own meal: 32.9%  Do handy work: 22.4%  Perform housework: 28%  Climb stairs: 36.6%  Manage medication: 35%  Manage own money: 23.5% |  |  |  |  |  |
| Mahmud NA *et al* (2020) | Malaysia | Cross-sectional | 2018 | 3977 | 68.3 | 52.9 | Older adults living in non-institutionalised living quarters from urban and rural areas of every state in Malaysia, as part of the National Health and Morbidity Survey 2018. Data from individuals aged ≥60 years were included in this study. | Barthel ADL Index | Prevalence of ADL limitation = 683/3977 (17.2%) | Prevalence of ADL limitation (n=683 out of 3977) was 17.2%, and IADL limitation (n=1925 out of 3977) was 48.4%. An estimated total of 547,881 individuals aged ≥60 years in Malaysia experienced limitation in at least one component of ADL, accounting for 17.0% (95% confidence interval [CI] = 15.0–19.2) of the total population. An estimated total of 1,334,111 individuals aged ≥60 years in Malaysia experienced limitations in at least one IADL, accounting for 42.9% (95%CI = 39.9–46.0) of the total population. | n/a | n/a | n/a | n/a |
|  |  |  |  |  |  |  |  | Lawton and Brody IADL Scale | [male, n=253; female, n=430] |  |  |  |  |  |
|  |  |  |  |  |  |  |  |  | Age 60-64: n=132 |  |  |  |  |  |
|  |  |  |  |  |  |  |  |  | Age 65-69: n=140 |  |  |  |  |  |
|  |  |  |  |  |  |  |  |  | Age 70-74: n=139 |  |  |  |  |  |
|  |  |  |  |  |  |  |  |  | Age 75-79: n= 127 |  |  |  |  |  |
|  |  |  |  |  |  |  |  |  | Age ≥80: n= 145 |  |  |  |  |  |
|  |  |  |  |  |  |  |  |  | Prevalence of IADL limitation = 1925/3977 (48.4%) |  |  |  |  |  |
|  |  |  |  |  |  |  |  |  | [male: n=745; female, n=1180] |  |  |  |  |  |
|  |  |  |  |  |  |  |  |  | Age 60-64: n=478 |  |  |  |  |  |
|  |  |  |  |  |  |  |  |  | Age 65-69: n=478 |  |  |  |  |  |
|  |  |  |  |  |  |  |  |  | Age 70-74: n=384 |  |  |  |  |  |
|  |  |  |  |  |  |  |  |  | Age 75-79: n=316 |  |  |  |  |  |
|  |  |  |  |  |  |  |  |  | Age ≥80: n=269 |  |  |  |  |  |
| Murat MF *et al* (2019) | Malaysia | Cross-sectional | 2013 | 258 | 66 ± 6.5 | 52.3 | Community-dwelling older adults aged 60 years and above from Mukim Batu, a semi-urban area in Gombak District, Selangor, Malaysia. | Lawton and Brody IADL questionnaire | IADL disability: n=150 (58.1%) (male, n=66 (53.7%), female, n=84 (62.2%)) | The prevalence of IADL disability among respondents was 58.1% (n=150). Prevalence was almost doubled for the older adults aged 75 years and above (male=89.5%; female=100%) compared to those aged 60 to 74 years (male=47.1%; female=58.9%). | n/a | n/a | n/a | n/a |
| Norazman CW *et al* (2020) | Malaysia | Cross-sectional | 2018-2019 | 301 | 67.08 ± 5.536 | 69.4 | Older adults aged 60 years and above who were residing in the People Housing Project (Projek Perumahan Rakyat) at Kuala Lumpur between October 2018–January 2019. | Lawton IADL scale-Malay version | Functional disability: n=155 (51.5%) | 51.5% (n=155) of the subjects were found to have functional disabilities. Female participants had a higher prevalence of functional disability (56.5%) than male participants (40.2%). | MMSE (Malay version) | Cognitive impairment: n=45 (15%) | The prevalence values of frailty and pre-frail from the study population were 15.9% and 72.8, The prevalence of poor cognitive function is at 15.0%. The incidence is higher among frail individuals. | n/a |
|  |  |  |  |  |  |  |  |  |  |  | Fried’s Frailty Phenotype | Frail: n=48 (15.9%) |  |  |
|  |  |  |  |  |  |  |  |  |  |  |  | Pre-frail: n=219 (72.8%) |  |  |
| Hamzah NAR *et al* (2018) | Malaysia | Cross-sectional | 2014-2015 | 269 | 69.5 ± 5.2 | 47.6 | Community-dwelling older adults aged 60 years and above living in three Federal Land Development Authority (FELDA) schemes in Johor. | Lawton IADL Scale | Dependent in IADL: n=128 (47.6%) | 47.6% of respondents (n=128) were dependent on performing IADL. Women reported higher dependency (63.3%) in performing items in the Lawton-IADL scale than men (30.8%). | Hodkinson Abbreviated Mental Test (HAMT) to assess cognitive function | Abnormal cognitive function [score 7 or less]: n=42 (15.6%) | Based on the SPPB questionnaire score, 30.9% of respondents had low performance, and this was more prevalent in women (33.8%) than men (27.7%). For cognitive function status, 15.6% of the respondents were classified as having an abnormal cognitive function (scores 7 or less). | n/a |
|  |  |  |  |  |  |  |  |  |  |  |  | Physical performance: |  |  |
|  |  |  |  |  |  |  |  |  |  |  |  | Low [score 0-6]: n=83 (30.9%) |  |  |
|  |  |  |  |  |  |  |  |  |  |  | Short Physical Performance Battery (SPPB) questionnaire to assess physical performance. | Intermediate [score 7-9]: n=123 (45.7%) |  |  |
| Safian N *et al* (2021) | Malaysia | Cross-sectional | 2018-2020 | 1024 | n/a | 57 | Community-dwelling older adults aged at least 60 years in urban and rural areas in Selangor. | Japan Gerontological Evaluation Study (JAGES) questionnaire: assistance in daily life | Limitations of activities in the past 6 months: | 41% of participants had limitations in daily activities within the past six months. The most impaired ADL was self-control for continence (3%), while, for IADL, it was the ability to fill a document by themselves (43%). 8% of the respondents needed assistance. | Self-declaration questions: sensory impairment (vision, hearing, mobility, and memory) | Difficulty seeing: n=833 (69.2%) | 69.2% and 26.9% of participants had difficulty seeing and hearing, respectively. Mobility difficulties (walking, climbing steps, carrying items) were present in 50.9% of participants. Difficulties in seeing, hearing, mobility, and memory or concentrating were significantly associated with the need for assistance. | n/a |
|  |  |  |  |  |  |  |  | 6-item ADL assessment (bathing, dressing, toilet use, transferring and self-control of continence, feeding). | Total: 41.1% (severe limited n=49, 4.1%; limited but not severe n=445, 37%) |  | Ability to climb stairs and walking: mobility and falls assessment | Difficulty hearing: n=324 (26.9%) |  |  |
|  |  |  |  |  |  |  |  |  | ADL dependency: |  |  | Difficulty walking, climbing steps and carrying items: n=613 (50.9%) |  |  |
|  |  |  |  |  |  |  |  |  | Bathing: n=10 (0.8%) |  |  |  |  |  |
|  |  |  |  |  |  |  |  |  | Dressing: n=10 (0.8%) |  |  |  |  |  |
|  |  |  |  |  |  |  |  |  | Toileting: n=9 (0.7%) |  |  |  |  |  |
|  |  |  |  |  |  |  |  |  | Transferring: n=28 (2.3%) |  |  |  |  |  |
|  |  |  |  |  |  |  |  | 7-item IADL assessment (able to go out alone, shopping, cook for themselves, pay bills, withdraw money, fill documents by themselves, and find a friend’s telephone number). | Feeding: n=5 (0.4%) |  |  |  |  |  |
|  |  |  |  |  |  |  |  |  | Poor self-control of continence: n=35 (2.9%) |  |  |  |  |  |
|  |  |  |  |  |  |  |  |  | IADL dependency: |  |  |  |  |  |
|  |  |  |  |  |  |  |  |  | Unable to go out alone: n=441 (36.6%) |  |  |  |  |  |
|  |  |  |  |  |  |  |  |  | Unable to go shopping: n=199 (16.5%) |  |  |  |  |  |
|  |  |  |  |  |  |  |  |  | Unable to cook for themselves: n=164 (13.6%) |  |  |  |  |  |
|  |  |  |  |  |  |  |  |  | Unable to pay bills: n=346 (28.7%) |  |  |  |  |  |
|  |  |  |  |  |  |  |  |  | Unable to withdraw money: n=438 (36.4%) |  |  |  |  |  |
|  |  |  |  |  |  |  |  |  | Unable to fill documents by themselves: n=512 (42.5%) |  |  |  |  |  |
|  |  |  |  |  |  |  |  |  | Unable to find friend’s telephone number: n=256 (21.3%) |  |  |  |  |  |
| Kua EH  (1990) | Singapore | Cross-sectional | 1985 | 1000 | n/a | 56 | Community-dwelling Chines older adults aged 65 years and above from three constituencies in Singapore. | 8-item ADL scale (shopping, walking, cooking, dressing, light chores (e.g., washing and sweeping), bathing, feeding and toileting). | Age 65-74: | 2.3% of the young-old (65-74) and 14.5% of the old-old (75 years and above) were moderately or severely impaired in ADL. | Geriatric Mental State Schedule (GMS) | Physical health: | The prevalence of dementia and depressive disorder was 1.8% and 4.6%, respectively. About 3% of young-old and 12.9% of old-old had moderate or severe physical health impairment. Comparing the two groups, the physical health of the old-old was more impaired (p<0.001). Of 253 participants with mild impairment, 14.5% had vision or hearing problems. | n/a |
|  |  |  |  |  |  |  |  |  | Mildly impaired [n=132, 30.6%], |  | Semi-structured questionnaire and physical examination | Young-old: 42.6% had minor disabilities; old-old: 38.3% had minor disabilities. |  |  |
|  |  |  |  |  |  |  |  |  | Moderately impaired [n=9, 2.1%], severely impaired [n=1, 0.2%] |  |  | 3% of young-old and 12.9% of old-old had moderate or severe impairment |  |  |
|  |  |  |  |  |  |  |  |  |  |  |  | Of the 253 participants (both young-old and old-old) with mild impairment, 14.5% had vision or hearing problems. |  |  |
|  |  |  |  |  |  |  |  |  | Age 75 and above: |  |  |  |  |  |
|  |  |  |  |  |  |  |  |  | Mildly impaired [n=96, 53.3%] |  |  |  |  |  |
|  |  |  |  |  |  |  |  |  | Moderately impaired [n=19, 10.6%] |  |  |  |  |  |
|  |  |  |  |  |  |  |  |  | Severely impaired [n=7, 3.9%] |  |  | Dementia prevalence: 1.8%. |  |  |
| Chan KM *et* (1999) | Singapore | Cross-sectional | 1992-1993 | 401 | n/a | n/a | Older persons aged 60 and older who live in the community in Singapore, recruited under the 'Community Study on State of Health, Function, Cognitive and Social State of Elderly People in Singapore' study. | Barthel ADL Index | 17.0% were dependent on at least one ADL | 68 subjects (17.0%) were dependent on at least one ADL. Female subjects and those 75 years old were more likely to be dependent. Forty subjects (10.4%) had at least 1 IADL function that they could not perform. The most common dependence in IADL was preparing a meal, shopping, taking their own medicine, and using the phone. The least frequent problem was in housekeeping. | n/a | n/a | n/a | n/a |
|  |  |  |  |  |  |  |  | 5-item IADL assessment (prepare a simple meal, shop for groceries, use the telephone independently, housekeeping and taking own medications). | Bowel (incontinence or occasional incontinence): n=11, 2.7% |  |  |  |  |  |
|  |  |  |  |  |  |  |  |  | Bladder (incontinence or occasional incontinence): n=58, 14.5% |  |  |  |  |  |
|  |  |  |  |  |  |  |  |  | Grooming (needs help): n=3, 0.8% |  |  |  |  |  |
|  |  |  |  |  |  |  |  |  | Toilet use (dependent or needs help): n=4, 1.0% |  |  |  |  |  |
|  |  |  |  |  |  |  |  |  | Feeding (unable to or needs help): n=2, 0.5% |  |  |  |  |  |
|  |  |  |  |  |  |  |  |  | Transfer (unable to, needs major help or needs minor help) n=6, 1.5% |  |  |  |  |  |
|  |  |  |  |  |  |  |  |  | Mobility (immobile or wheelchair independent or needs untrained help) n=7, 1.7% |  |  |  |  |  |
|  |  |  |  |  |  |  |  |  | Dressing (dependent or needs help) n=2, 0.5% |  |  |  |  |  |
|  |  |  |  |  |  |  |  |  | Steps (unable to or needs help) n=9, 2.2% |  |  |  |  |  |
|  |  |  |  |  |  |  |  |  | Bathing (dependent) n=2, 0.5%. |  |  |  |  |  |
|  |  |  |  |  |  |  |  |  | 10.4% had at least 1 IADL difficulty |  |  |  |  |  |
|  |  |  |  |  |  |  |  |  | Meals: n=22, 5.6% |  |  |  |  |  |
|  |  |  |  |  |  |  |  |  | Shopping: n=19, 4.8% |  |  |  |  |  |
|  |  |  |  |  |  |  |  |  | Take medicine: n=15, 3.8% |  |  |  |  |  |
|  |  |  |  |  |  |  |  |  | Phone: n=19, 4.8% |  |  |  |  |  |
|  |  |  |  |  |  |  |  |  | Housekeeping: n=15, 3.8% |  |  |  |  |  |
| Malhotra *et al* (2012) | Singapore | Cross-sectional | 2009 | 5000 | n/a | 54.2 | Community-dwelling Singaporeans aged 60 years and over, from the Social Isolation, Health and Lifestyles Survey (SIHLS) 2009. | 7-item ADL scale (bathing, dressing, eating, toileting, standing/sitting, walking (around the house), and going outside). | Overall prevalence of ADL limitations: 9.7% [women:12.7%; men:6.1%] | The overall prevalence of ADL limitations was 9.7%. Prevalence was higher in women (12.7%) than in men (6.1%) and among those 75 years and older compared with those aged 65-74 years. The ADL with the highest prevalence of limitation was going outside, followed by bathing, walking, standing/sitting, dressing, toileting and eating. | n/a | n/a | n/a | n/a |
|  |  |  |  |  |  |  |  |  | Total prevalence: |  |  |  |  |  |
|  |  |  |  |  |  |  |  |  | Limitation in: |  |  |  |  |  |
|  |  |  |  |  |  |  |  |  | Bathing: 4.5% |  |  |  |  |  |
|  |  |  |  |  |  |  |  |  | Dressing: 3.8% |  |  |  |  |  |
|  |  |  |  |  |  |  |  |  | Eating: 1.5% |  |  |  |  |  |
|  |  |  |  |  |  |  |  |  | Toileting: 2.5% |  |  |  |  |  |
|  |  |  |  |  |  |  |  |  | Standing up/sitting down: 3.9% |  |  |  |  |  |
|  |  |  |  |  |  |  |  |  | Walking: 4.4% |  |  |  |  |  |
|  |  |  |  |  |  |  |  |  | Going outdoors: 9.0% |  |  |  |  |  |
| Merchant RA *et al* (2017) | Singapore | Cohort study | 2015-2016 | 1051 | 71.2 | 57.2 | Healthy Older People Everyday (HOPE) study involving community-dwelling older adults aged 65 years and older from a defined geographical area in the Northwest region of Singapore. | Barthel Index | ADL ≥1 impairment: | A significantly larger proportion of frail participants had impairment in 1 or more ADL or IADL than prefrail and robust participants. The proportion of robust, prefrail, and frail participants with one or more ADL impairment was 9.2%, 28.3% and 47.4%, respectively. The proportion of robust, prefrail, and frail participants with one or more IADL impairment was 25%, 35.5% and 47.7%, respectively. | FRAIL (Fatigue, Resistance, Ambulation, Illness, and Loss of Weight) scale for frailty assessment | Frailty prevalence= 6.2% | The prevalence of frailty and prefrailty was 6.2% and 37%, respectively. The prevalence of frailty increased from 5.4% in the 65- to 79-year-olds to 14.0% in the 80 years and older group. | n/a |
|  |  |  |  |  |  |  |  | Lawton IADL scale | Robust: n=55 (9.2%) |  | MMSE to assess cognitive function | Cognitive Impairment: |  |  |
|  |  |  |  |  |  |  |  |  | Prefrail: n=110 (28.3%) |  |  | Robust: n=32 (5.5%) |  |  |
|  |  |  |  |  |  |  |  |  | Frail: n=31 (47.7%) |  |  | Prefrail: n= 40 (10.3%) |  |  |
|  |  |  |  |  |  |  |  |  | IADL ≥1 impairment: |  | Physical performance screening: Timed-Up-and-Go (TUG) test and handgrip strength | Frail: n=13 (20.0%) |  |  |
|  |  |  |  |  |  |  |  |  |  |  |  | Total: n=85 (8.1%) |  |  |
|  |  |  |  |  |  |  |  |  | Robust: n=148 (25%) |  |  | Low handgrip strength: |  |  |
|  |  |  |  |  |  |  |  |  | Prefrail: n=138 (35.5%) |  |  | Robust: n=199 (44.0%) |  |  |
|  |  |  |  |  |  |  |  |  | Frail: n=31 (47.7%) |  |  | Pre-frail: n=141 (49.3%) |  |  |
|  |  |  |  |  |  |  |  |  |  |  |  | Frail: n=28 (65.1%) |  |  |
| Ng TP *et al* (2006) | Singapore | Cross-sectional | 2003-2004 | 1079 | 69.6 ± 7.4 | 54 | Analysis was based on data collected from the National Mental Health Survey of the Elderly, Singapore, a population-based survey of a nationally representative sample of non-institutionalised older adults aged 60 and older. | Modified Barthel Index | Prevalence of 10-item ADL dependence: 10.8% | The population-weighted prevalence of ADL dependency (needing personal assistance in at least one of the 10 ADLs of the Barthel Index) was 10.8% (95% CI 9.0-12.7%). The prevalence of functional disability based on at least one item of the five ADLs was lower (5.0%). The overall prevalence of functional disability increased with advancing age. The greatest proportions of participants were disabled in climbing stairs (9.6%), walking inside the house (4.4%), and transferring from bed/chair (3.6%), difficulties that reflect lower torso functional limitations. These were followed by bathing (3.0%), grooming (2.4%), dressing (2.3%), bladder control (2.2%), toileting (1.7%), bowel control (1.6%), and feeding (1.1%). | MMSE for cognitive assessment | 23.2% (n=248) had MMSE test scores of 23 or less | Percentage of participants with severe (MMSE score <19) or mild to moderate cognitive impairment (MMSE scores 19-23) was 5.6% and 17.6%, respectively. Dementia was present in 5.5% of participants. | n/a |
|  |  |  |  |  |  |  |  | Katz ADL scale (feeding, bathing, dressing, toileting, transferring) | Prevalence of functional disability based on at least one item of the five ADLs: 5.0% |  |  |  |  |  |
|  |  |  |  |  |  |  |  |  | MMSE score <19 (severe); Prevalence of ADL dependence: 49.2% |  |  |  |  |  |
|  |  |  |  |  |  |  |  |  | MMSE score 19-23 (mild-moderate); Prevalence of ADL dependence: 20.0% |  |  |  |  |  |
|  |  |  |  |  |  |  |  |  | Hearing impairment; Prevalence of ADL dependence: 33.0% |  |  |  |  |  |
|  |  |  |  |  |  |  |  |  | Visual impairment; Prevalence of ADL dependence: 32.2% |  |  |  |  |  |
| Nyunt MS *et al [35]* (2012) | Singapore | Cross-sectional | n/a | 267 | 72.5 ± 7.9 | 58.8 | Older persons aged 60 and above with clinically significant depressive symptoms were identified among participants in the Community-Based Early Psychiatric Intervention Strategy (CEPIS) program in Singapore. | Barthel ADL Index | ADL status: | The proportion of ADL-independent participants at 12-months follow-up was 61.8% compared to 73% at baseline. The proportion of participants who were IADL-independent at 12-months follow-up was 15% compared to 24% at baseline. Remission of depression had a positive impact in improving ADL functioning, but it did not appear to be associated with a positive impact on IADL. | Cognitive functioning of the participants was assessed at baseline using MMSE. The MMSE (cut-off: 23/24) is used to differentiate those with and without dementia | n/a | n/a | n/a |
|  |  |  |  |  |  |  |  | Lawton and Brody IADL scale | Independent in all ADL at baseline: 73% |  |  |  |  |  |
|  |  |  |  |  |  |  |  |  | Independent in all ADL at 12-month follow-up: 61.8% |  |  |  |  |  |
|  |  |  |  |  |  |  |  |  | IADL status: |  |  |  |  |  |
|  |  |  |  |  |  |  |  |  | Independent in all IADL at baseline: 24.0% |  |  |  |  |  |
|  |  |  |  |  |  |  |  |  | Independent in all IADL at 12-month follow-up: 15.0% |  |  |  |  |  |
| Quah JHM *et al* [38] (2017) | Singapore | Cross-sectional | 2014 | 498 (participants with multimorbidity) | 73.9 ±6.5 | 49 | Older adults aged 65 years or older with multimorbidity and who were on regular follow-up and seeking medical attention for chronic medical condition(s) only. Participants were recruited from a primary care clinic (SingHealth Polyclinics-Outram) in Singapore. | Modified Barthel Index | ADL dependency: | For individual ADL, the most and least common problems were bowel or bladder control (17.7%) and toileting (1.4%), respectively. For individual IADL, the most and least common problems were housekeeping (11.6%) and telephone (2.6%). Almost 75% of the older adults with multimorbidity were “independent” and “needs no help”. | n/a | Physical disability: n=35 (7%) | n/a | An increased number of dependent ADL and IADL dimensions were strongly associated with worse health-related quality of life. |
|  |  |  |  |  |  |  |  | Lawton and Brody IADL Scale | Feeding, bathing, grooming/dressing: n=9 (1.8%) |  |  |  |  |  |
|  |  |  |  |  |  |  |  |  | Bowel/bladder control: n=88 (17.7%) |  |  |  |  |  |
|  |  |  |  |  |  |  |  |  | Toileting: n=7 (1.4%) |  |  |  |  |  |
|  |  |  |  |  |  |  |  |  | Transfer, mobility/climbing stairs: n=59 (11.8%) |  |  |  |  |  |
|  |  |  |  |  |  |  |  |  | IADL dependency: |  |  |  |  |  |
|  |  |  |  |  |  |  |  |  | Telephone: n=13 (12.6%) |  |  |  |  |  |
|  |  |  |  |  |  |  |  |  | Shopping: n=50 (10%) |  |  |  |  |  |
|  |  |  |  |  |  |  |  |  | Food preparation: n=47 (9.4%) |  |  |  |  |  |
|  |  |  |  |  |  |  |  |  | Housekeeping: n=58 (11.6%) |  |  |  |  |  |
|  |  |  |  |  |  |  |  |  | Laundry: n=42 (8.4%) |  |  |  |  |  |
|  |  |  |  |  |  |  |  |  | Public transport: n= 51 (10.2%) |  |  |  |  |  |
|  |  |  |  |  |  |  |  |  | Medication: n=35 (7%) |  |  |  |  |  |
|  |  |  |  |  |  |  |  |  | Finance: n=38 (7.6%) |  |  |  |  |  |
| Tan TL *et al* (2001) | Singapore | Retrospective observational study | 1998 | 78 | n/a | 46.15 | Older patients attending the Geriatric Assessment Clinic (GAC) at Tan Tock Seng Hospital were evaluated with emphasis on the presence of geriatric syndromes (falls, impaired immobility, incontinence, impaired cognition). | Data were extracted from case records. | 55% of the patients with impaired mobility had associated decline in at least one BADL, with nearly a third (31%) having four or more BADL declines (13%, 5%, 6%, 19% and 12% had a decline in one, two, three, four and all five BADLs, respectively). | 55% of the patients with impaired mobility had associated decline in at least one BADL. Under each category of BADL, transfer, dressing, toileting or bathing were equally affected. | Mobility status was based on patients’ subjective descriptions and physicians’ assessment. | Mobility impairment: n=78 (50%) | Impaired mobility was the presenting complaint in 67.9% of the patients. For the remaining 32.1%, impaired mobility was identified as one of the problems after assessment. Less than half (35 patients, 45%) could ambulate independently at presentation. Fifteen (19%) required supervision in ambulation, 14 (18%) required assistance, and the last 14 (18%) were chair bound or bed bound. | Impairment in mobility greatly impacts mortality, morbidity, and functional capacity of older persons. This would translate to an enormous socio-economic burden in the care of these patients, mainly when impaired mobility was closely associated with other geriatric syndromes. |
|  |  |  |  |  |  |  |  | Clinical protocol regularly recorded functional status of attending patients, details of mobility status, duration of mobility decline and BADL status; mobility status reassessed after short-term follow-up (4 months). | Under each category of BADL, transfer, dressing, toileting or bathing were equally affected (35%, 34%, 31% and 36%, respectively), while the decline in self-feeding was found in 19% of patients. |  |  | Less than half n=35 (45%) can ambulate independently at presentation. |  |  |
|  |  |  |  |  |  |  |  |  |  |  |  | N=15 (19%) required supervision |  |  |
|  |  |  |  |  |  |  |  |  |  |  |  | n=14 (18%) required assistance |  |  |
|  |  |  |  |  |  |  |  |  |  |  |  | n=14 (18%) were chair or bed-bound |  |  |
| Tareque MI *et al* (2019) | Singapore | Cross-sectional | Longitudinal survey in 2009 (wave 1), 2011 (wave 2) and 2015 (wave 3). | 3452 | 70.1, 75 | 53.3, 55.2 | Data were from a nationally representative longitudinal survey, Panel on Health and Ageing of Singaporean Elderly (PHASE), that collected information on community-dwelling Singaporeans aged 60 years or older in 2009 (wave 1), 2011 (wave 2), and 2015 (wave 3). | 6-item ADL (bathing; dressing; eating; standing up from bed/chair or sitting down on a chair; walking indoors; toileting) 7-item IADL (preparing own meals; shopping; managing money; using the telephone; doing housework; taking transportation; taking prescribed medication) | Prevalence of ADL limitation (BADL and IADL) across three waves: 13.9% to 21.1% | Across three waves, the prevalence of limitation in ADLs (ADL and IADL) ranged from 13.9% to 21.1%, respectively. Those older, women and those with primary or less education were more likely to have both ADL and physical impairments versus no or either impairment. | Self-reported question on vision impairment | Visual impairment: 12% to 17% | The prevalence of limitation in physical function ranged from 34.5% to 50.9%. At each wave, the lowest prevalence of limitation in physical function and ADLs was observed among those with no vision or hearing impairment and the highest among those with both impairments. Across waves, 12% to 17% of older adults had vision impairment, 6% to 9% had hearing impairment, and 9% to 13% had both impairments. A substantial proportion, 34.6% from wave 1 to 2 and 42.7% from wave 2 to 3, experienced a change in their vision and hearing impairment status between consecutive waves. | Older adults with visual and hearing impairments are expected to live more years with physical and ADL limitations. |
|  |  |  |  |  |  |  |  |  |  |  | Self-reported question on hearing status | Hearing impairment: 6% to 9% |  |  |
|  |  |  |  |  |  |  |  |  |  |  | Limitation in physical function (walking 200-300 m; climbing ten steps without resting; standing/going without sitting for 2 hours; continuous sitting for 2 hours; stooping or bending knees; raising hands above the head; extending arms out in front as if to shake hands; grasping with fingers or moving fingers easily, and lifting an object weighing 5 kg) | Physical function: 34.5% to 50.9% |  |  |
| Yong V *et al* (2011) | Singapore | Cross-sectional | 2005 | 2134 (aged 65 years and above) | n/a (for desired age group) | 52.4 | Data were from the 2005 National Survey of Senior Citizens (NSSC) in Singapore, a nationally representative survey of Singapore citizens and permanent residents aged 55 and over. | BADL scale (bathing, dressing, toileting, transferring, and feeding). | Functional disability | The prevalence of having at least one ADL disability was higher among the 75 and above group compared to the 65-74 age group. Differences in functional disabilities by gender are the most apparent at the higher ages of 75 years and above, with a larger proportion of women experiencing more of all disability items—bathing, dressing, toileting, transferring, and feeding difficulties compared to men. 17% of women reported having at least one disability versus 10% of men. Among the activities of daily living, bathing and dressing present the most, and feeding presents the least difficulties at older ages. | Participants were interviewed to assess their hearing, vision and mobility problems. | Vision impairment: | Prevalence for vision impairment in the 65-74 age group was 15.16% for males and 16.17% for females, while for the 75 and above age group, it was 20.44% for males and 26.51% for females. Prevalence for hearing impairment in the 65-74 age group was 6.85% for males and 6.67% for females, while for the 75 and above age group, it was 18.08% for males and 18.2% for females. Prevalence for walking impairment in the 65-74 age group was 6.18% for males and 8.29% for females, while for the 75 and above age group, it was 17.48% for males and 26.35% for females. | The gender differences in years of life with disabilities increase with age. For all activities of daily living items, women can expect a higher proportion of life with disabilities than men. The largest gender difference is for bathing and eating, where women can expect about 10% of their remaining lives (about twice that of men’s) with these difficulties. |
|  |  |  |  |  |  |  |  |  | (65-74 years old) |  |  | 65-74: |  |  |
|  |  |  |  |  |  |  |  |  | Males (n=639) |  |  | Male: 15.16% |  |  |
|  |  |  |  |  |  |  |  |  | Bathing: 2.55% |  |  | Female: 16.17% |  |  |
|  |  |  |  |  |  |  |  |  | Dressing: 2.56% |  |  | 75+: |  |  |
|  |  |  |  |  |  |  |  |  | Toileting: 2.41% |  |  | Male: 20.44% |  |  |
|  |  |  |  |  |  |  |  |  | Transferring: 2.42% |  |  | Female: 26.51% |  |  |
|  |  |  |  |  |  |  |  |  | Feeding: 1.28% |  |  |  |  |  |
|  |  |  |  |  |  |  |  |  | At least 1 ADL: 2.70% |  |  | Hearing impairment |  |  |
|  |  |  |  |  |  |  |  |  | Female (n=701) |  |  | 65-74: |  |  |
|  |  |  |  |  |  |  |  |  | Bathing: 3.20% |  |  | Male: 6.85% |  |  |
|  |  |  |  |  |  |  |  |  | Dressing: 3.35% |  |  | Female: 6.67% |  |  |
|  |  |  |  |  |  |  |  |  | Toileting: 2.96% |  |  | 75+: |  |  |
|  |  |  |  |  |  |  |  |  | Transferring: 2.96% |  |  | Male: 18.08% |  |  |
|  |  |  |  |  |  |  |  |  | Feeding: 1.30% |  |  | Female: 18.20% |  |  |
|  |  |  |  |  |  |  |  |  | At least 1 ADL: 3.88% |  |  | Walking impairment: |  |  |
|  |  |  |  |  |  |  |  |  | (75+) |  |  | 65-74: |  |  |
|  |  |  |  |  |  |  |  |  | Males (n=331) |  |  | Male: 6.18% |  |  |
|  |  |  |  |  |  |  |  |  | Bathing: 6.91% |  |  | Female: 8.29% |  |  |
|  |  |  |  |  |  |  |  |  | Dressing: 6.45% |  |  | 75+: |  |  |
|  |  |  |  |  |  |  |  |  | Toileting: 6.12% |  |  | Male: 17.48% |  |  |
|  |  |  |  |  |  |  |  |  | Transferring: 6.7% |  |  | Female: 26.35% |  |  |
|  |  |  |  |  |  |  |  |  | Feeding: 3.03% |  |  |  |  |  |
|  |  |  |  |  |  |  |  |  | At least 1 ADL: 9.78% |  |  |  |  |  |
|  |  |  |  |  |  |  |  |  | Females (n=463) |  |  |  |  |  |
|  |  |  |  |  |  |  |  |  | Bathing: 14.23% |  |  |  |  |  |
|  |  |  |  |  |  |  |  |  | Dressing: 14.49% |  |  |  |  |  |
|  |  |  |  |  |  |  |  |  | Toileting: 12.91% |  |  |  |  |  |
|  |  |  |  |  |  |  |  |  | Transferring: 12.63% |  |  |  |  |  |
|  |  |  |  |  |  |  |  |  | Feeding: 6.97% |  |  |  |  |  |
|  |  |  |  |  |  |  |  |  | At least 1 ADL: 16.82% |  |  |  |  |  |
| Boongird C *et al* (2011) | Thailand | Cross-sectional | 2007-2008 | 177 | 75 ± 7 | 67 | Patients from one geriatric assessment clinic at the family medicine outpatient clinic of Ramathibodi Hospital. | Modified ADL Barthel Index | Either ADL or IADL functionally dependent: 28.5% | 25.4% of patients had partial dependence on ADL or IADL, and 3.1% had total dependence on ADL. | History of hearing problem | Visual acuity of more than 20/60 in either eye: 81.5% | The percentage of participants with MMSE scores of 24 or less and clock drawing test scores of 0 were 63% and 50.6%, respectively. The three most common geriatric syndromes were dementia/MCI (60.4%), gait problems (30.5%) and depression (29.9%). 81.5% reported impairment in visual acuity of more than 20/60 in either eye. | n/a |
|  |  |  |  |  |  |  |  | Modified IADL Chula Index |  |  | Snellen eye chart | Audition impairment: 23% |  |  |
|  |  |  |  |  |  |  |  |  |  |  | Cognition: Thai MMSE and clock drawing test | MMSE score <24: 64% |  |  |
|  |  |  |  |  |  |  |  |  |  |  | Dementia: DSM-IV and National Institute of Neurological Disorder and Stroke and Association Internationale pour Recherche et l’Enseignement en Neuroscience (NINDS-AIREN) criteria | Clock drawing score 0: 50.6% |  |  |
| Jiawiwatkul U *et al* (2012) | Thailand | Cross-sectional | 1997-2004 | 4048 persons in 1997 | n/a | 57.2 (1997), 51.4 (2004) | Prevalence of disability in ADLs was obtained from the second and third National Health Examination Surveys (NHES II and NHES III), conducted in 1997 and 2004, respectively. The NHES II and NHES III are cross-sectional surveys of multistage probability sampling of the non-institutionalised Thai population. The present study focused on older people aged 60 years and older. | 6-item ADL scale (eating, dressing, bathing, toileting, moving within the house, and moving between bed and chair). | Prevalence of disability among older men decreased from 9.8% in 1997 to 3.6% in 2004, whereas that of older women decreased from 13.0% in 1997 to 5.2% in 2004. Older women had a significantly higher prevalence of ADL disabilities in almost every age group than older men. | The age-standardised prevalence of disability among older men decreased from 9.8% in 1997 to 3.6% in 2004, whereas older women decreased from 13.0% in 1997 to 5.2% in 2004. Older women had a significantly higher prevalence of ADL disabilities in almost every age group than older men. Those with lower education had a higher prevalence of disabilities. There was a reduction in the disability rate during the 7-year period. | n/a | n/a | n/a | n/a |
|  |  |  |  | 19372 persons in 2004 |  |  |  |  |  |  |  |  |  |  |
| Jitapunkul S *et al* (2000) | Thailand | Cross-sectional | 1997 | 4048 | 69.7 ± 7.3 | 57 | People aged 60 years and above from the National Health Examination Survey II 1997. | Modified Barthel ADL Index | Prevalence rates of self-care disability: 6.9% | Prevalence rate of self-care ADL disability was 6.9% (96% CI 6.1-7.7). Percentage of participants with mild, moderate or severe ADL severity were 4.3%, 1.2% and 1.4%, respectively. Those in the 80 years and above age group had the highest prevalence of dependency in any ADL (19.1%). | n/a | n/a | n/a | Although women have a longer life expectancy than men, they spend more years in disabled states. |
|  |  |  |  |  |  |  |  | Self/carer-reported need for assistance in ADL (feeding, grooming, transferring, toileting, dressing and bathing) | Mild: 4.3% (n=174) |  |  |  |  |  |
|  |  |  |  |  |  |  |  |  | Moderate: 1.2% (n=49) |  |  |  |  |  |
|  |  |  |  |  |  |  |  |  | Severe: 1.4% (n=57) |  |  |  |  |  |
|  |  |  |  |  |  |  |  | *mild: dependent on 1-2 ADLs; |  |  |  |  |  |  |
|  |  |  |  |  |  |  |  | *moderate: dependent on 3-4; |  |  |  |  |  |  |
|  |  |  |  |  |  |  |  | *Severe: dependent on five or more ADLs |  |  |  |  |  |  |
| Praditsuwan R *et al* (2012) | Thailand | Prospective cohort study | 2009 | 225 | 78 ± 5.9 | 49.3 | Patients aged 70 or older admitted to general medical wards from January to March 2009 at the Department of Medicine, Faculty of Medicine Siriraj Hospital, Mahidol University, Thailand. | 10-item Barthel Index | Number of participants with data on impairment=193 | Out of 193 patients with data on impairment, the proportion of patients with impaired ADL was 59.6%, and impaired IADL was 89.6%. | Thai MMSE | Visual impairment: 85.1% (n=137) total number 0f participants=161 | Dementia was present in 41.8% (n=94 out of 225). Visual impairment was present in 85.1% (n=137 of 161), and hearing impairment was present in 5.5% (n=10 of 183). | n/a |
|  |  |  |  |  |  |  |  | 5-item Lawton and Brody IADL scale | ADL disability: 59.6% (n=115) |  | Initial assessment with patient interview, proxy interview and review of medical record | Hearing Impairment: 5.5% (n=10) total number of participants=183 |  |  |
|  |  |  |  |  |  |  |  |  | IADL disability: 89.6% (n=173) |  |  |  |  |  |
| Prasitsiriphon O *et al [37]* (2019) | Thailand | Cross-sectional | 2009 | 8272 | 64.1 (younger female population), 73.9 (older female population) | 50.9 | The study population was drawn from the 2009 National Health Examination Survey of Thailand (NHES IV), with a representative sample of the non-institutionalized population. The sample population was restricted to respondents aged 60 to 79 years in this study. | Modified Katz ADL scale | ADL disabilities: | The prevalence of ADL and IADL disabilities in younger females (60-69 years) was 1.4% and 56%. The older (70-79 years) group had a higher prevalence of limitations (3% ADL disabilities, 81.3% IADL disabilities). For males, the prevalence of ADL and ADL disabilities was 1.2% and 32.9%, respectively in the younger age (60-69 years) group, and 3.7% and 52.8% respectively in the older age (70-79 years) group. | Questions-based memory assessment: concentration and learning | Cognitive impairment: | In the older age group, males and females had a higher prevalence of cognitive impairment, upper-body and lower-body limitations, and higher functional limitations than their younger counterparts. Inability to perform tasks of daily living is more likely to increase with increasing weakness in muscular strength than IADL tasks or other functional limitations. | n/a |
|  |  |  |  |  |  |  |  | 5-item IADL assessment (using the telephone, handling money, managing medication, using transportation or driving, and housekeeping) | Male |  | Upper and lower body limitations | Male |  |  |
|  |  |  |  |  |  |  |  |  | 60-69 years: 1.2% |  | Higher functional limitation | 60-69 years: 5.5% |  |  |
|  |  |  |  |  |  |  |  |  | 70-79 years: 3.7% |  | Overall function based on handgrip strength and usual walking speed | 70-79 years: 9.1% |  |  |
|  |  |  |  |  |  |  |  |  | Female |  |  | Female |  |  |
|  |  |  |  |  |  |  |  |  | 60-69 years: 1.4% |  |  | 60-69 years: 11.2% |  |  |
|  |  |  |  |  |  |  |  |  | 70-79 years: 3% |  |  | 70-79 years: 17.2% |  |  |
|  |  |  |  |  |  |  |  |  | IADL disabilities: |  |  |  |  |  |
|  |  |  |  |  |  |  |  |  | Male |  |  |  |  |  |
|  |  |  |  |  |  |  |  |  | 60-69 years: 32.9% |  |  |  |  |  |
|  |  |  |  |  |  |  |  |  | 70-79 years: 52.8% |  |  |  |  |  |
|  |  |  |  |  |  |  |  |  | Female |  |  |  |  |  |
|  |  |  |  |  |  |  |  |  | 60-69 years: 56% |  |  |  |  |  |
|  |  |  |  |  |  |  |  |  | 70-79 years: 81.3% |  |  |  |  |  |
| Taboonpong S *et al [42]* (2008) | Thailand | Cross-sectional | n/a | 400 | n/a | 59.8 | Individuals aged 60 years and above from a province with 14 districts in southern Thailand. | Modified Chula IADL Index | IADL score: | The percentage of subjects with inactivity in IADL was 52.1% (n=195) | Cognition: Chula Mental Test (CMT) | Cognitive impairment (score 0-14): n=60 (15%) | 15% of subjects had cognitive impairment (CMT scores 0-14). Significant predictors of cognitive impairment were found to include inactivity in relation to the IADLs, no formal education, female gender, and depression. | Impairment of IADL could be a consequence of cognitive decline and vice versa. |
|  |  |  |  |  |  |  |  |  | 0-7 (inactive): n=195 (52%) |  |  |  |  |  |
|  |  |  |  |  |  |  |  |  | 8-10 (active): n=179 (47.9%) |  |  |  |  |  |
| Thiamwong L *et al [48]* (2017) | Thailand | Cross-sectional | 2010 | 386 | 71.11 ± 7.73 | 64.5 | Older adults aged 60 years and over who lived in rural communities in a province of Southern Thailand. | Barthel ADL scale | ADL | For both non-fear of falling and fear of falling groups, the combined proportion of those needing some or complete help was highest for bathing and toileting. Fear of falling had significant relationships with perceived general health, visual impairment, mobility impairment, balance impairment, stroke, hypertension, antihypertensive drug, number of medications, history of falls, and activities of daily living. | Snellen chart was used to assess visual acuity | Visual impairment: n=153 (39.63%) | Combined prevalence for both participant groups for visual, mobility and balance impairment were 39.63%, 14.8% and 25.1%. | Participants with balance impairments were over three times more likely to have a fear of falling than those without balance impairments. Fear of falling can lead older adults to restrict activity beyond the level warranted by physical injuries resulting from the fall itself. |
|  |  |  |  |  |  |  |  |  | Eating |  |  |  |  |  |
|  |  |  |  |  |  |  |  |  | some help: n=171 (44.3%) |  |  |  |  |  |
|  |  |  |  |  |  |  |  |  | complete help: n=1 (0.3%) |  | Mobility based on a) being bed-bound; b) being homebound; c) being able to walk outside with assistive device or physical assistance from others; d) able to walk outside independently. | Mobility impairment: n=57 (14.8%) |  |  |
|  |  |  |  |  |  |  |  |  | Bathing |  |  | Balance impairment: n=97 (25.1%) |  |  |
|  |  |  |  |  |  |  |  |  | some help: n=253 (65.5%) |  |  |  |  |  |
|  |  |  |  |  |  |  |  |  | complete help: n=6 (1.6%) |  |  |  |  |  |
|  |  |  |  |  |  |  |  |  | Dressing |  |  |  |  |  |
|  |  |  |  |  |  |  |  |  | some help: n=164 (42.49%) |  |  |  |  |  |
|  |  |  |  |  |  |  |  |  | complete help: n=1 (0.26%) |  |  |  |  |  |
|  |  |  |  |  |  |  |  |  | Toileting |  |  |  |  |  |
|  |  |  |  |  |  |  |  |  | some help: n=230 (59.59%) |  |  |  |  |  |
|  |  |  |  |  |  |  |  |  | complete help: n=6 (1.55%) |  | For balance impairment, participants were asked to perform full tandem tested balance. |  |  |  |
|  |  |  |  |  |  |  |  |  | Walking |  |  |  |  |  |
|  |  |  |  |  |  |  |  |  | some help: n=187 (48.4%) |  |  |  |  |  |
|  |  |  |  |  |  |  |  |  | complete help: n=5 (1.3%) |  |  |  |  |  |
| Nguyen TV *et al* (2021) | Vietnam | Prospective cohort study | 2016-2017 | 180 | 80.6±8.2 | 50 | Patients aged 65 years or older with chronic heart failure admitted to Thong Nhat Hospital (Cardiology Department and Interventional Cardiology Department) in Ho Chi Minh City. | Katz ADL scale | The activity that was most commonly impaired was bathing (21.1%), followed by transferring (20.0%), toileting (12.2%), dressing (8.9%), eating (3.3%) and continence (2.8%) | Overall, participants with ADL impairment were significantly older, had a higher hospitalisation rate in the past year, and had a higher prevalence of stroke. The activity that was most commonly impaired was bathing (21.1%), followed by transferring (20.0%), toileting (12.2%), dressing (8.9%), eating (3.3%) and continence (2.8%). There was no significant difference between men and women. | n/a | n/a | n/a | ADL impairment was significantly associated with an increased likelihood of readmission (unadjusted OR 3.75, 95% CI 1.87 to 7.53). This association was examined further in multivariate logistic regression, adjusted to age, sex and the variables that had a significant relationship with readmission on univariate analyses. After adjusting to age, sex, history of hospitalisation in the past year, the total number of comorbidities, anaemia and chronic kidney disease, the impact of ADL impairment on 3-month readmission was still significant (adjusted OR 2.75, 95% CI 1.25 to 6.05, p=0.01). |
| Nguyen TTH et al 2021 | Vietnam | Cross-sectional | 2016 | 251 | 84.6 + 4.2 | 68.5 | People aged 80 years and over living in 5 communes in Soc Son district, a suburban area in northern Hanoi. | Lawton and Brody Scale | The most commonly impaired activity was telephone use (70.9%) and cooking (64.1%). | The prevalence of functional disability (disability with ≥3 IADL items) was 64.5%. The most commonly impaired activity was telephone use (70.9%) and cooking (64.1%). | Fried’s frailty phenotype | n/a | Among 251 participants, 28 (11.2%) were frail and 127 (50.6%) prefrail. | n/a |
|  |  |  |  |  |  |  |  |  | Prevalence of IADL: 162/251 (64.5%) |  |  |  |  |  |
| Vinh Nguyen T *et al* (2020) | Vietnam | Cross-sectional | 2019 | 695 | 71.4 | 57.4 | People aged 60 years in urban and rural communities in Thanh Hoa city and Hoang Hoa district of Thanh Hoa province, Vietnam. | Katz ADL Index | ADL disability: 11.6% | BADL and IADL disability prevalence was 11.6% and 15.6%, respectively. The prevalence of both BADL and IADL was higher in those with worse self-reported health, more than two chronic conditions, hearing impairment cognitive impairment. Living in rural areas was associated with BADL disability while increasing age was associated with IADL disability. | Self-reported visual and hearing impairment | Visual impairment: n=403 | 64.9% of rural participants and 49.3% of urban participants reported visual impairment. Regardless of where the participants lived, nearly 80% had chronic diseases, 15% had hearing loss, and 26% had cognitive impairment. | n/a |
|  |  |  |  |  |  |  |  | Lawton IADL scale | IADL disability: 15.6% |  |  | Hearing impairment: n=108 |  |  |
|  |  |  |  |  |  |  |  |  | Visual impairment with ADL disability: 16.9% |  |  | Cognitive impairment: n=179 |  |  |
|  |  |  |  |  |  |  |  |  | Visual impairment with IADL disability: 20.8% |  | Vietnamese version MMSE |  |  |  |
|  |  |  |  |  |  |  |  |  | Hearing impairment with ADL disability: 32.0% |  |  |  |  |  |
|  |  |  |  |  |  |  |  |  | Hearing impairment with IADL disability: 40.2% |  |  |  |  |  |
|  |  |  |  |  |  |  |  |  | Cognitive impairment with ADL disability: 31.6% |  |  |  |  |  |
|  |  |  |  |  |  |  |  |  | Cognitive impairment with IADL disability: 39.7% |  |  |  |  |  |

Note: ADL: Activities of daily living, BADL: Basic activities of daily living, CMT: Chula Mental Test, GMS: DSI: dual sensory impairment, Geriatric Mental State Schedule, HAMT: Hodkinson Abbreviated Mental Test, IADL: Instrumental activities of daily living, MCI: Mild Cognitive Impairment, MMSE: Mini-Mental State Examination, SPPB: Short Physical Performance Battery, TUG: Timed Up and Go test, VI: visual impairment, n/a: not available.
