# Supplementary material for: The prevalence of functional disability and its impact on older adults in the ASEAN region: a systematic review and meta-analysis
Source: Epidemiol Health. 2022 Jul 12;44:e2022058. doi: 10.4178/epih.e2022058 (PMC9754909; doi:10.4178/epih.e2022058)
Supplement: Supplementary Material 4. — Quality assessment for included studies using the adapted Newcastle Ottawa Scale. [file epih-44-e2022058-suppl4.docx]

Supplementary Material 4. Quality assessment for included studies using the adapted Newcastle Ottawa Scale.

| **Studies included** | **Selection** | | | **Comparability** | | **Outcome** | | **Total (maximum 10 stars)** |
| --- | --- | --- | --- | --- | --- | --- | --- | --- |
|  | **Representativeness of the sample** | **Sample size** | **Non-respondents** | **Ascertainment of the exposure (risk factor)** | **Comparability of study** | **Assessment of outcome** | **Statistical test** |  |
| Pengpid S *et al* (2020) | ★ | ★ | ★ | ★★ | ★★ | ★★ | ★ | 10 |
| Rensa R *et al* (2019) | ★ | ★ |  | ★★ |  | ★ | ★ | 6 |
| Setiati S *et al* (2019) |  | ★ |  | ★★ |  | ★★ | ★ | 6 |
| Soejono CH *et al* (2019) | ★ | ★ | ★ | ★★ |  | ★★ | ★ | 8 |
| Nambooze J *et al* (2014) | ★ | ★ |  | ★★ |  | ★ | ★ | 6 |
| Falahaty K *et al* (2015) | ★ | ★ |  | ★ | ★ | ★ | ★ | 6 |
| Hairi NN *et al* (2010) | ★ | ★ | ★ | ★★ | ★★ | ★★ | ★ | 10 |
| Harithasan D *et al* (2020) | ★ | ★ |  | ★★ | ★★ | ★★ | ★ | 9 |
| Loh KY *et al* (2005) | ★ | ★ |  | ★ |  | ★ | ★ | 5 |
| Mahmud NA *et al* (2020) | ★ | ★ | ★ | ★★ | ★★ | ★ | ★ | 9 |
| Murat MF *et al* (2019) | ★ | ★ | ★ | ★★ | ★★ | ★ | ★ | 9 |
| Norazman CW *et al* (2020) | ★ | ★ |  | ★ ★ | ★ | ★ | ★ | 7 |
| Hamzah NAR *et al* (2018) | ★ | ★ |  | ★★ |  | ★ | ★ | 6 |
| Safian N *et al* (2021) | ★ | ★ | ★ | ★★ | ★★ | ★ | ★ | 9 |
| Kua EH (1990) | ★ | ★ |  | ★★ |  | ★ |  | 5 |
| Chan KM *et al* (1999) | ★ | ★ |  | ★★ |  | ★ | ★ | 6 |
| Malhotra *et al* (2012) | ★ | ★ |  | ★ | ★★ | ★ | ★ | 7 |
| Merchant RA *et al* (2017) | ★ | ★ | ★ | ★★ | ★★ | ★★ | ★ | 10 |
| Ng TP *et al* (2006) | ★ | ★ |  | ★★ | ★★ | ★ | ★ | 8 |
| Nyunt MS *et al* (2012) | ★ | ★ |  | ★★ | ★★ | ★★ | ★ | 9 |
| Quah JHM *et al* (2017) | ★ | ★ |  | ★★ | ★★ | ★ | ★ | 8 |
| Tan TL *et al* (2001) | ★ | ★ |  | ★ |  | ★★ | ★ | 6 |
| Tareque MI *et al* (2019) | ★ | ★ |  | ★ | ★★ | ★ | ★ | 7 |
| Yong V *et al* (2011) | ★ | ★ |  | ★ |  | ★ | ★ | 5 |
| Boongird C *et al* (2011) | ★ | ★ | ★ | ★★ |  | ★★ | ★ | 8 |
| Jiawiwatkul U *et al* (2012) | ★ | ★ | ★ | ★ | ★★ | ★ | ★ | 8 |
| Jitapunkul S *et al* (2000) | ★ | ★ | ★ | ★★ | ★★ | ★ |  | 8 |
| Praditsuwan R *et al* (2012) | ★ | ★ |  | ★★ |  | ★ | ★ | 6 |
| Prasitsiriphon O *et al* (2019) | ★ | ★ |  | ★★ | ★★ | ★★ | ★ | 9 |
| Taboonpong S *et al* (2008) | ★ | ★ | ★ | ★★ | ★★ | ★★ | ★ | 10 |
| Thiamwong L *et al* (2017) | ★ | ★ |  | ★★ |  | ★★ | ★ | 7 |
| Nguyen TV *et al* (2021) | ★ | ★ |  | ★ | ★★ | ★ | ★ | 7 |
| Nguyen TTH et al (2021) | ★ | ★ |  | ★ | ★ | ★ |  | 5 |
| Vinh Nguyen T *et al* (2020) | ★ | ★ | ★ | ★★ | ★★ | ★ | ★ | 9 |
